# Conceptualizing COVID-19 and Public Panic with the Moderating Role of Media Use and Uncertainty in China: An Empirical Framework

**DOI:** 10.3390/healthcare8030249

**Published:** 2020-08-02

**Authors:** Tao Xu, Usman Sattar

**Affiliations:** College of Law and Political Science, Zhejiang Normal University, Jinhua 321004, China

**Keywords:** novel coronavirus, anxiety, cognition, psychosocial analysis, news reporting, stress management, public health

## Abstract

Uncertainty puts people in a binary state of mind, where every piece of external information can positively or negatively affect their state of health. Given the uncertain situation created by the new coronavirus pandemic, this study claims to be the first empirical analysis of the real-time status of public panic in China. It frames peoples’ intrinsic and extrinsic stimuli, creating a psychosocial analysis of public panic. We conducted an online survey of WeChat and QQ users in February 2020 and collected 1613 samples through a QR code questionnaire. We used the ordinary least squares (OLS) regression equation model to conceptualize public panic pathways in different gender and age groups. This underlines the psychological origins of fear and anxiety and points out how the media uses socially constructed public panic. The results show that the outbreak of COVID-19 created uncertainty among the public, and the official media intensified it because of the late dissemination of news about the outbreak’s real-time status. Hence, unofficial media remained faster in news reporting, but the news reporting remained contradictory with official reports. This created doubts about the authenticity of the given information and caused public mental health abnormalities. The study provides a conceptual framework based on lessons learned from physiology, psychology, and social psychology and real-time public analysis to inform policymakers and public administrators about the contextual dynamics of public panic in China. It provides useful insights into the wise handling of this uncertain time and controlling the fatal conditions of public panic created by COVID-19. It has implications for other countries as well.

## 1. Introduction

The new coronavirus disease, termed COVID-19 by the World Health Organization (WHO) [1,2,3], has left people in a state of mind such that fear of the unknown has severely affected the mental well-being of people of all ages worldwide [4,5,6]. With the emergence of this deadly virus, people are locked down wherever they are. It has caused people to lose their source of income [7], and they are lagging behind in the necessities of life [8,9]. Hence, social distancing, as a key prevention method to stay away from this virus, has further damaged their emotions [10]. Complex emotions such as fear, panic, anxiety, sadness, etc., have a significant physiological basis and psychological abnormalities, and are influenced by the psychosocial environment [11,12,13]. Although the latter subject areas have yet to be categorically studied with regard to the sources of panic, a coherent framework keeping in view the outbreak of COVID-19 is urgently needed to guide policymakers and public administrators in ensuring public mental health [14,15,16]. Therefore, this study first retrieves the core theoretical viewpoints of panic from physiology, psychology, and social psychology, and tests two hypotheses based on given thoughts, and finally provides a framework for the conceptual path of public panic in the era of COVID-19.

### 1.1. Existing Theoretical Perspectives of Panic

This section reviews the cross-disciplinary strands of panic, including the subject matter of physiology, psychology, and social psychology.

#### 1.1.1. Perspective of Physiological Analysis

According to the biological view of the study of panic, fear is closely related to brain structure. People’s complex emotions such as anger, panic, sadness, happiness, and disgust activate the brain’s amygdala and the reactive state of mind changes based on those emotions. Therefore, the amygdala and the gray matter around the midbrain aqueduct change correspondingly with the swing of emotions [17]. Stimulus information is passed through the sensory organs into the thalamus and corresponding cortex, and then into the lateral amygdala. After being processed by the complex inner amygdala circuit, it passes by the relevant nerve nucleus in the hypothalamus and brainstem by the amygdala’s central nucleus, regulating the body’s physiological and behavioral responses to fear stimuli. This is the response mechanism of the human brain region where fear occurs.

Darwin found that the mechanism of emotional change is not limited to humans, but widely exists in non-human primates such as mammals, rodents, and even invertebrates. He proposed the famous hypothesis of interspecies consistency, that there is a high degree of functional and behavioral similarity in the expression of fear, disgust, and aggression among different species [18]. It might be innate. Some scholars believe that this kind of inborn fear comes from the fear of predators. From this standpoint, anxiety can be simply divided into two categories in terms of how it is acquired: innate and learned. Innate fear is the fear of predators and aggressive conspecifics. This kind of fear is encoded in the genes. The typical Pavlov experiment can explain learned fear; that is, the emotional response of fear is produced by a terrifying stimulus.

To figure out the difference between these two kinds of fear, some scholars have studied their neural mechanisms. Gross and Canteras divided fear into three types: fear toward predators, toward conspecifics’ aggressive behavior, and toward reflexive learning of pain stimulus. Correspondingly, they summarized three neural pathways for each of these three types of fear [19].

Davis et al. proposed a working model for the central nucleus of the amygdala (CeA) and the bed nucleus of the stria terminalis (BNST) to explain different types of fear on time scales based on how panic and anxiety arise. According to this working model, fear-related stimuli first rapidly activate the basolateral amygdala (BLA) and medial CeA to trigger a rapid panic response. On the other hand, lateral CeA inputs release corticotropin-releasing factor (CRF) to the BNST and trigger a slower and more prolonged anxiety response. The function of inhibitory feedback from the BNST can also terminate the panic response, thus completing the smooth switch from panic to anxiety [20].

Mobbs et al. [21] introduced the concept of a predator threat continuum in ecology into human FMRI (Functional Magnetic Resonance Imaging)research. He thought that when the threat is abstract or spatially distant, it can correspond to the post-encounter phase, and when the threat is extremely close, it can correspond to the predator’s circa-strike phase. Thus, he devised a series of experiments to study how the brain works when humans are in these two kinds of states, and when they switch between them.

#### 1.1.2. Perspective of Psychological Analysis

The above studies are of great significance to comprehend the biological basis of individual fear. However, panic cannot be fully interpreted only by biological or neurological explanations. Fear in humans does not stay at the same level as fear in animals. Compared with animals, humans have stronger processing power, better memory, a clear ability to express themselves through language, and the ability to think logically. Therefore, human fear not only comes from heredity and genetics, but is also stimulated through social interactions, society, and culture. This base further promotes the study of panic from a psychological viewpoint.

From the psychological perspective, panic is regarded as a group emotion or mentality that can be generated through threats faced or imagined by humans. It is an essential adaptive function in the process of survival and evolution [22,23]. Many scholars believe that threat is an important factor that causes fear. For example, Basoglu et al. [24] believe that when people encounter natural disasters, they will have negative psychological reactions, such as feeling insecure, worry, anxiety, and fear. Armfield notes that the higher the degree of danger, the stronger the response of fear [25].

On the psychological level, looking at where fear as a group emotion comes from, it is found that a high-risk perception of things is closely connected with the psychology of fear, and the sense of uncontrollability brought by high risk is a crucial reason for people to fear certain things. In addition, fear is closely related to people’s sense of unpredictability and uncertainty toward things [26]. The degree of fear is not only relevant to the danger brought by the subject itself, but is also relevant to an individual’s perception and cognition of the danger. As different individuals have different experiences, knowledge, and other aspects, their cognition and interpretation of things or information may not be entirely consistent. Hence, there is a certain degree of difference in fear among different individuals. Scholars such as Graziano et al. and King et al. [23,27] found that demographic variables such as age, gender, geographical location, and socioeconomic status have a significant impact on the differences of fear [23,27].

Studying panic as an emotional reaction toward external stimuli brings a new perspective and field, thus creating the era of stress theory. From the standpoint of social psychology, panic is a psychological response to external stimuli. Following this concept, Wei et al. summarized three models of stress theory [28].

The stimulus theory model of stress regards all stimuli that cause tense responses in individuals as stress and the consequences of external stimuli as reactions. The emphasis is to try to find causal relations, even quantitative relationships, between stimuli and tension responses. However, this theoretical model ignores the complexity of people’s subjective initiative and psychological behavior. It is impossible to determine the stimulus factor that causes tension in the same individual in all occasions. Thus, the theory is inadequate to establish a quantitative relationship between the intensity of tension stimulus (stress) and the response level of tension.

The second model is the response theory model of stress. With regard to the dilemma in seeking a causal relationship between the stress response (the result) and the stimulus factor, people act in a diametrically opposite way. They first identify and describe the stress response (the effect), and then demonstrate the irritability of the corresponding stimulus (cause). However, the stress response model still regards humans as living entities reacting passively to the adverse environment without seeing their attitudes and behavior under stress.

In fact, these two theories both explore the relationship between stress and reaction, but their logic is different. The former holds that there is stimulation first, and then there is a result, while the latter explores the result first and then finds the possible stimulus source. Both of them ignore the role of the environment in the stress and reaction.

Thus, the cognition-phenomenology-transaction (CPT) theory model of stress is noteworthy here. This is a theoretical model that involves more stress psychology and behavioral processes. This model contains five aspects: stimulus, primary appraisal, secondary appraisal, and the interactional result of individual environmental effects. It holds that stress is caused by specific relationships between individuals and the environment. If people think that they are unable to cope with the environmental demands, they will have a stressful experience. It emphasizes the interaction between individuals and the environment, pays attention to the subjective initiative of individuals in the stressful situation, and sees the significance of information feedback and behavioral adjustment.

With the above theories, many scholars have studied the panic caused by disaster events from different angles. Concerning individual sensitivity factors, Langford et al. proposed a social cognitive psychological model to predict individual risk perception and preference [29]. Slovic et al. put forward three basic dimensions of public perception of risk: “fear risk,” “unknown risk,” and “the number of individuals facing a particular risk.” [30] Fischhoff proposed a model of psychological noise [31]. Some scholars took the SARS epidemic as an example to study public risk perception, public panic behavior, and emergency response strategies [32,33,34]. Some, such as Li [35], took the Wenchuan earthquake as an example to study public panic and the response to sudden disasters. Some compared public psychological states and characteristics in various regions and different degrees of disasters [33,36]. Some of them also paid attention to the connection between news dissemination and panic during major epidemics [36,37,38].

### 1.2. Critical Assessment of the Given Perspectives

Stress theories are developed based on physiology and later carried forward through psychology, which provides us with new ideas to explain a complex human emotion (panic). Nevertheless, whether we consider stimulus theory, response theory, or CPT theory, they all hold that panic is a behavioral response following stimulation by stressors. However, panic not only can exist in human genes and be learned from external stimuli, but also can be constructed from the external social environment. This means that the study of panic also needs to consider the analysis of external social forces. Panic not only can be learned but also can be constructed. A very small number of studies focus on media communication in major disasters, and most of them almost ignore the social construction of group panic. Therefore, this study fills this gap by examining the source of public panic in the era of COVID-19. From the perspective of social construction, we analyze how public panic is constructed, besides the substantial pandemic threat, and how it is deepened in the process of secondary (distorted) transmission of information related to the pandemic in the news media (including wemedia). Thus, we point out two generative paths and mechanisms of group panic: the path of stimulus–cognition, and the path of information dissemination–social construction.

### 1.3. New Analytical Framework and Hypothesis

Theoretically speaking, the main reason for group tension, anxiety, and panic is that people are at a higher level of uncertainty and feel insecure. With the fear of the unknown, people cannot judge how long the crisis will last. They are in a state of mind with no clear picture of what might happen to them at any moment. The feeling of being unsafe and the fear of possible imminent crisis in their lives makes them feel panicky. Similarly, people in China do not know when the virus will completely vanish. 

In terms of daily life experience in China, since Wuhan in Hubei Province was closed on 23 January due to COVID-19, the first or second response mechanism for the virus was activated in different regions. The whole society was in an uncertain state of mind regarding the level at which they would be able to combat COVID-19. All kinds of enterprises, companies, shopping malls, restaurants, and service industries were suddenly shut down, and public movement was strictly restricted. Public administrators did their best, but the transmission and infection of COVID-19 spread rapidly in almost all provinces of China. The morale of most people was down, and they felt scared. With these warning signs for others, people rushed to purchase various anti-epidemic materials, such as masks, disinfectants, goggles, and protective clothes. When most people realized that they should wear masks in daily life to avoid the spread of COVID-19, they found that they could not buy any type of mask or alcoholic disinfectant from pharmacies, supermarkets, or even online. This experience left people in great uncertainty. As COVID-19 is a sudden and emerging infectious disease, even doctors and scientists know little about it. Therefore, the fight against the virus ultimately depends on the individual’s immune system to reduce the spread of the virus in one’s own body. The enormous risk in everyday life, as well as the limited medical awareness and treatment of the virus, places people in great uncertainty. They do not know who is already infected around them, or whether they would recover in case of infection or beat the virus and survive. The uncertainty in their knowledge of the virus and their experience of daily life makes them feel panic.

In addition, in view of the experience and lessons of Hubei Province and Wuhan city, cities have taken various countermeasures to block the chain of transmission of the virus, including blocking traffic, closing villages and communities, and other active measures. These active measures have produced positive effects in terms of blocking the transmission of the virus, but also generated significant negative effects. First of all, local governments issued policies to extend the holiday and delay the return to work. This caused a shortage of social service items such as medical supplies and daily necessities, which were already restricted during the Spring Festival, but it got even worse. The high-risk characteristics of the airborne and human-to-human transmission of the virus led the public to switch to online shopping.

However, in the short term, due to the sudden increase in online orders, the online platforms were inadequate to meet the huge distribution demand. Many families were unable to get their orders delivered successfully. Meanwhile, most offline shopping places were closed for epidemic prevention, and only a few supermarkets were still open to supply groceries, and a considerable flow rate of people in supermarkets increased the risk of contact and transmission of the virus. Consequently, they were stuck in places where they did not have the basics of life. This increased the uncertainty and panic. To this end, we propose Hypothesis 1.

**Hypothesis** **1.** *The greater the uncertainty among people, the more panic they feel.*

In terms of information dissemination, a lack of data about the actual numbers of confirmed cases, suspected cases, deaths, isolated cases, and related information about the real-time situation, if not spread accurately and timely, will lead to public suspicion, doubt, and panic. Hence, if real-time data are available but not published timely and accurately, it also puts people into a state of panic. Truthful reporting helps the public to understand the relevant situation and alerts them in a timely manner, thus enhancing their protection and avoiding a larger transmission of the virus. In the early days of the pandemic, many contradictory articles in the media led people to doubt even the valid information. They also doubted the official data because of the prevailing rumors. Though the number of infected cases was increasing every day, factual reporting could have helped the public understand the real-time progress and adopt relevant measures and strategies in response according to the directions given by the government. Moreover, they could also have had relatively stable psychological expectations, so as not to be in extreme distrust and panic.

Besides, if the official information does not meet the acceptable standards, the public seeks information through unofficial channels. These informal ways of providing information through social media or other online sources proceeded with the first information about the pandemic. Thus, people were more inclined to look forward to these channels of information. This was also the reason for all kinds of gossip and rumors. Unofficial channels spread a great deal of false, inaccurate, distorted, or even fabricated information. These eye-catching sources are sometimes engaged in commercial gain rather than promoting public health. Whatever the case, the widespread dissemination of such grapevine news aggravated people’s judgment of the outbreak and gratuitously created panic that should not have occurred at any cost. It made the previous nervous and panicky crowd even more fearful.

On the contrary, timely disclosure of information by official channels can keep people informed of the status of the outbreak, and can stabilize relevant psychological expectations and correct the inaccurate, distorted, and twisted parts of the information transmitted through the grapevine. It allows people to fully understand accurate information, thus helping them to avoid groundless panic. To this end, we propose Hypothesis 2.

**Hypothesis** **2.** *The influence of uncertainly on the extent of people’s panic is moderated by channels of information.*

**Figure 1:**
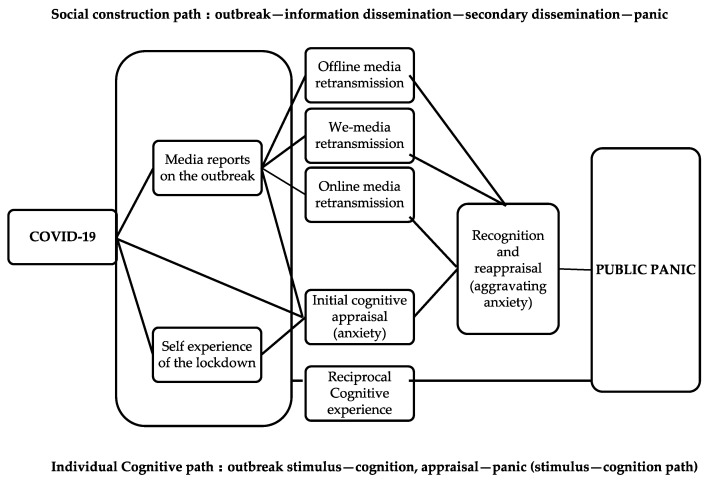
Conceptual framework.

Therefore, it seems that not only can people be frightened by outside stimuli and feel panic, but cognitive stimulation, activated by the spread of secondary information, also generates panic. In other words, panic can be learned as well as be constructed. Based on this idea, to analyze group panic from the perspective of society, we should not only apply the existing social psychological analysis perspective of stimulus and cognition, but also introduce the sociocultural analysis perspective of information dissemination and social construction (Figure 1).

## 2. Materials and Methods

### 2.1. Data, Variables, and Measurements

#### 2.1.1. Data

This study collected data through a web survey, Public Perception of COVID-19 and Its Social Consequences in 2020, which was mainly aimed at understanding people’s perception of COVID-19 and its consequences after the outbreak of the epidemic. We generated a QR code of the questionnaire through WeChat in February 2020, then released the QR code in research group members’ WeChat and QQ friend circles and further disseminated it through these circles, i.e., the snowball method [39,40]. It took nearly a week, and eventually we obtained 1613 cases, covering almost all regions in northeastern, northern, eastern, central, southern, southwestern, and northwestern China. There are two reasons why we adopted this method. First, it enabled us to collect relevant information in a very short period of time, so as to timely reflect the impacts of the COVID-19 outbreak. Second, a more important concern in this study is the relationship between the information spread by wemedia such as WeChat and microblogs and public panic. Collecting relevant information through the Internet can accurately reflect the relevant situation of Internet users, which helps us to explore the relationship between them.

After deleting 76 missing surveys because more than one-third of questions were not answered, we used 1537 responses. Although many respondents are acquaintances through WeChat and QQ, the samples obtained through the chain sampling method are very inclusive and adequately representative, with high heterogeneity. As the respondents filled out the survey without any financial reward, the data are authentic. Although this kind of online survey has certain limitations in terms of representativeness due to its overall ambiguity, it still has practical significance given the current situation of the COVID-19 outbreak. All data were processed by SPSS 16.0 (SPSS Inc., Chicago, U.S.)and Stata 13.0 (StataCorp., TX, U.S.).

#### 2.1.2. Dependent Variable

The questionnaire included a set of subjective evaluation variables that scale psychological emotions, including doubt, tension, worry, helplessness, trepidation, sadness, fear, etc., with answers ranging from very inconsistent to very consistent in 5 intervals (see Supplementary Materials Table S1). Through aggregation, we attained the dependent variable. Specifically, in the analysis, the correlation of variables was first investigated and internal consistency was checked, and then exploratory factor analysis was done, and only one factor was extracted according to the internal consistency: we called it public panic.

**Figure 2:**
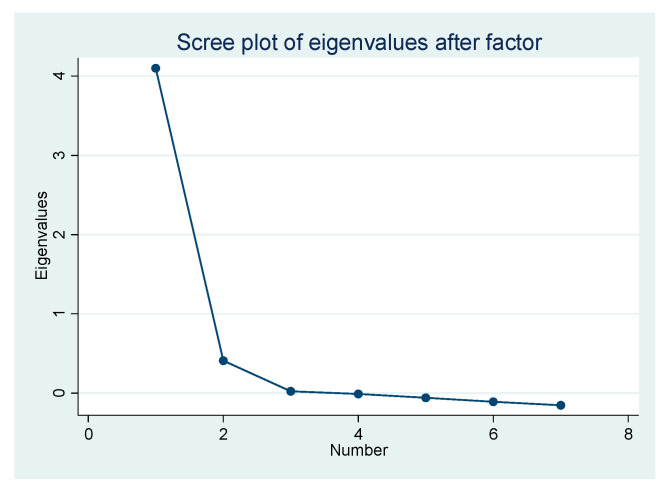
Scree plot of eigenvalues after factor.

We first tested the correlation of the emotional variables’ indicators and found that the indicators were closely related. The test result of reliability was alpha = 0.9035, and the Kaiser–Meyer-Olkin value was 0.8738 (*p* < 0.000), indicating that these indicators were suitable for exploratory factor analysis. Therefore, we conducted exploratory factor analysis by principal component and varimax rotation. From the results of Table 1 and Figure 2, we can see that only the eigenvalue of factor 1 is larger than 1, which shows that only one factor can be extracted. So, we extracted the factor and called it panic according to the correlation of indicators and their meanings. The factor score was saved as the dependent variable.

In order to estimate the outcome of public panic, we used several questions on people’s subjective feelings as the dependent variables of consequences of the panic model. Questions were asked regarding the consistency of respondents’ experiences, such as poor sleep, recalling the outbreak repeatedly, accelerated heartbeat and tension, and recurrent nightmares (see Supplementary Materials Table S2). The answers ranged from very inconsistent to very consistent in 5 intervals, coded as 1 to 5.

#### 2.1.3. Independent Variable and Major Explanatory Variable

Uncertainty usually refers to the inability to accurately know the result of a certain thing or decision in advance. In this study, it refers to people’s inability to accurately understand the epidemic itself and its consequences, and its prevention and control. Therefore, people’s uncertainty is reflected by their cognition of the epidemic situation itself, the consequences of the epidemic, and the government’s preventing and controlling it. We use these three groups of variables to reflect people’s uncertainty. The specific procedure for the three groups of variables is as follows.

Social cognition of the virus is represented by a set of subjective estimations, including high infectivity, rapid fatality, high mortality, and the lack of a specific drug to cure it, taken as an explanatory variable. The variables are measured on an ordinal scale with 6 possible answers: strongly disagree, disagree, somewhat disagree, somewhat agree, agree, and strongly agree, coded as 1 to 6.

Cognition of the consequences brought by the outbreak is based on respondents’ subjective judgment, which is measured on a 5-level scale, from completely uncontrollable to completely controllable. For the perception of the risk of the outbreak itself, the answers are complete natural risk, mixture of natural and human-made risk, and complete human-made risk, with scores from 1 to 3 assigned during analysis.

Concerning the external situation of the outbreak, the survey mainly checked on the frequency of attention paid to social media. The answers on the degree of attention ranged from very little concern to very much concern on a 5-level scale. The answers on frequency of attention ranged from not concerned, once every few days, once a day, twice a day, 3–5 times a day, and more than 5 times a day, scored from 1 to 5. The respondents’ views on further expansion of the epidemic outbreak, concern about information transparency, and concern about adequate life materials were also on a 5-level scale, ranging from totally disapprove to totally approve, scored from 1 to 5.

Media use refers to the platforms used by respondents to obtain information related to the outbreak. Answers included Xinhua news agency, China Central Television (CCTV), People’s Daily mainstream media, traditional newspapers, magazines, official microblogs, official accounts, commercial accounts, microblogs, WeChat circles, personal microblogs, Douyin, Kuaishou, and other short videos, relatives, friends, colleagues, etc. Official TV stations, newspapers, official microblogs, and official accounts are taken as official media, and personal microblogs, WeChat, Douyin, or individuals are considered as unofficial media.

People’s feelings about the government’s preventing and controlling the situation were measured on a 5-level scale of very bad, bad, not bad and not good, good, and very good. People’s feelings about the government’s efficient response were also measured on 5-level scale of very low efficiency, low efficiency, neither low nor high efficiency, high efficiency, and very high efficiency.

#### 2.1.4. Control Variable

The nominal variables include gender (male coded as 0, female coded as 1), and area (northeast, north, east, central, south, southwest, and northwest China). We also had a second way to code areas. In order to show the difference between Hubei Province, which was the first place to have a COVID-19 outbreak, and other provinces, we coded Hubei as 1 and other areas as 0. Education was measured as an ordinal variable (primary school or below, junior highschool, highschool, college, master’s degree, or above). Age was measured as a ratio variable, and age squared was created as a control variable.

#### 2.1.5. Model

This paper used the linear regression equation model [41,42] and the ordinary least squares (OLS) method [43] to analyze public panic. The specific expression is as follows:

Y = β_0_ + β_1_X_1_ + β_2_X_2_ + … + β_k_X_n_ + ε (1)

where Y is the dependent variable, panic, X represents various independent variables that may affect panic, and β is the relative influence coefficient.

Then the ordinal logistic regression model [44,45] was used to estimate the outcomes of panic. The model is as follows:

(2) $$\ln\frac{P\left(Y\leq j\right)}{1-p\left(Y\leq j\right)}=a_{j}+\beta_{1}X_{1}+\beta_{2}X_{2}+\ldots +\beta_{n}X_{n}$$

In this model, a_j_ represents thresholds, β represents parameters, and X is a set of factors or predictors.

## 3. Results

### 3.1. Manifestation and Characteristics of Panic

In terms of people’s emotions during the outbreak, the average score for feeling confused is 3.41, for feeling nervous is 3.52, for feeling worried is 3.75, for feeling helpless is 2.95, for feeling Trepidation is 2.91, for feeling sadness is 3.15, and for feeling fear is 2.93 (Table 2). In general, people’s negative emotions are more obvious. However, the scores of emotions like worry are the highest, while the scores of emotions like panic are relatively low. This indicates that people’s negative emotions are mainly dominated by worry, and although panic exists to a certain extent, it is not the main emotive reaction.

Looking at the age distribution of negative emotions, there is no significant age difference in all kinds of emotional reactions among people younger the age of 60; however, people older than 60 are not that worried by the outbreak. There is no evidence of negative feelings, worry, or panic among people over 60; however, all other age groups are significantly affected.

**Figure 3:**
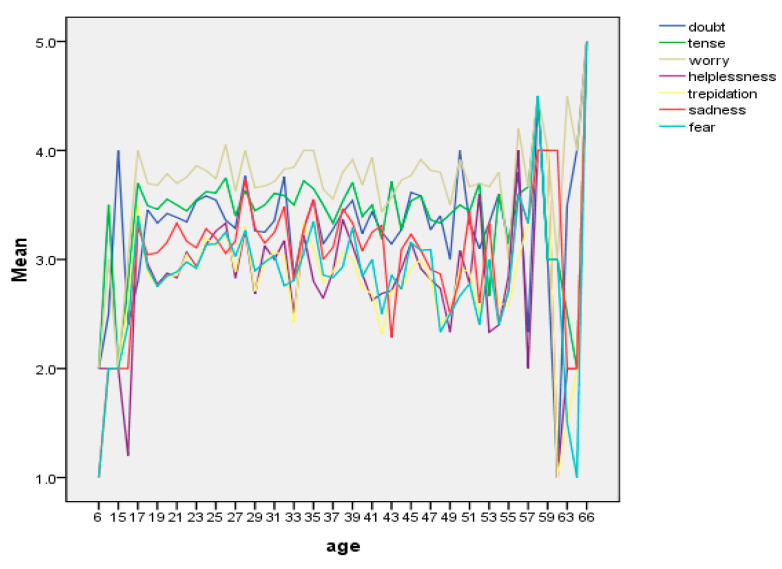
Mean panic rate with age.

Although people of all ages are commonly susceptible to COVID-19, the fatality rate of young adults is relatively low (Figure 3). At the same time, that of the elderly is relatively high, and especially the elderly with coronary heart disease, hypertension, hyperlipidemia, and other diseases have the highest fatality rate. The elderly feel more panicked by COVID-19 than the young and middle-aged groups. As a result, some older people are more worried and panicky. On the other hand, older people older than 60 have gone through the wind and rain of life and have seen great storms. They have a relatively peaceful state of mind to accept the consequences. We noticed that they were relatively calm and did not show obvious panic. The two groups have opposite mindsets, so the trend chart of data shows an up-and-down wave pattern.

**Figure 4:**
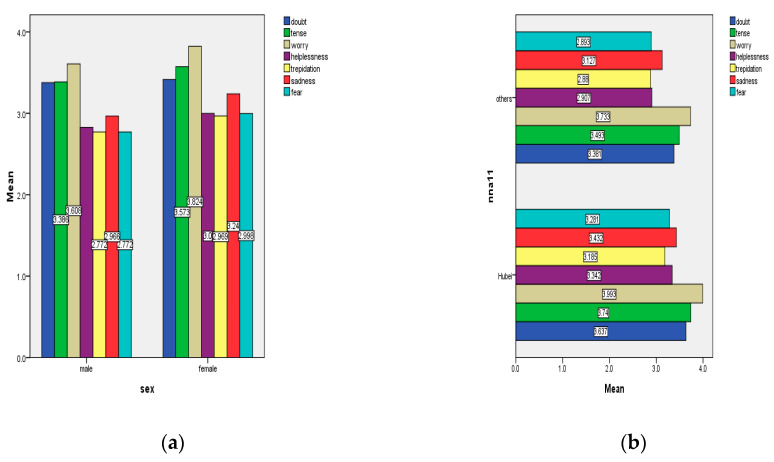
Comparison of panic between (**a**) different genders and (**b**) people in Hubei and other provinces in China.

In terms of gender, the average scores for panic among women are higher than for men in almost every emotional aspect, suggesting that women experience more negative emotional shocks than men. It shows that women are more sensitive, while men are comparatively less so. Men are relatively calmer than women (Figure 4a). In terms of the region, people in Hubei Province have significantly higher levels of panic than those in other provinces in China (Figure 4b). This may be closely related to the fact that people in Wuhan were the first to encounter this virus in China.

### 3.2. Uncertainty, Media Use, and Panic

#### 3.2.1. Cognition of the Virus Itself, Consequences, Attention, and Panic

According to cognition of the novel coronavirus (model 1), knowledge of the high mortality rate of COVID-19 and the lack of a specific medicine to cure it prominently increased the public’s anxiety and negative emotions (Table 3). Once the effects on the novel coronavirus are added (model 2), the cognition effects on the high mortality of COVID-19 are eliminated. However, the perception of the high infectivity of the novel coronavirus and the unavailability of specific drugs notably increased the level of anxiety among people. This suggests that public anxiety and panic about the novel coronavirus primarily comes from the negative consequences for individuals and society. The most terrible thing is the high infectivity rate and no specific drug for treatment. This has significantly increased the feeling of anxiety among people. If people considered it as a controllable disease, they might be relatively calm. Besides, the perception that COVID-19 is a natural risk has caused panic, and the impression of the risk of contracting it socially further intensifies the panic. This indicates that the social perception of risk also significantly affects people’s panic.

The novel coronavirus has attracted a lot of attention because of its high infection rate. According to the results of model 3, the more attention people give it and the more frequent the attention, the deeper their feelings of worry and panic. In the process of paying attention to the outbreak, if the outbreak is more widespread, people will be more worried and panicky. On the contrary, if the information about the outbreak is more transparent, people’s worry and panic will reduce. If people doubt the transparency of the outbreak information, their worry and panic will increase. The above results confirm hypothesis 1, that there is a strong positive correlation between uncertainty and people’s cognition.

#### 3.2.2. Moderating Role of Media and Panic

Model 4 examines the relationship between people’s approach to obtaining information about COVID-19 and their emotions of panic. After incorporating the approach to obtaining information, the original cognitive variables of the novel coronavirus itself (high infectivity, no specific drug, etc.) still prominently affect people’s panic. The negative consequences of COVID-19 for individuals and society still intensify people’s panic, and the expansion of the outbreak scope and the perception of whether COVID-19 is controllable are still important variables increasing people’s panic. Finally, there is also a strong link between the approach to obtaining information and people’s panic.

The results of model 4 also show that people tend to confirm the information about COVID-19 from both official and unofficial media (wemedia), and the more information people get, the more panic they feel. This is exactly in line with the projected situation. The data collection time (February) for this survey was at the peak of COVID-19 in China, and news on the infection and transmission situation was the most widely spread information in both official media and wemedia every day. At that time, there was no sign that the spread of COVID-19 was under control. Thus, obtaining information from multiple sources further increased people’s emotions of panic. Therefore, the use of both official media and wemedia is both positively correlated with people’s emotions of panic.

However, there is a clear difference between official media and wemedia in the acquisition and dissemination of information. In the process of releasing information, official media outlets have stricter confirmation, censorship, and other rules to ensure authenticity. The unofficial media is relatively loose in the course of information acquisition and dissemination. However, this relatively loose environment enables wemedia to release all kinds of information faster. This loose environment leads to numerous unverified rumors spreading through wemedia in the form of information. Wemedia, especially commercial channels with a broad audience, often do not have an intrinsic goal to disseminate information. The fundamental purpose is to acquire a large audience. A large audience means business value, and business value can be transformed into commercial profit. Plenty of commercial wemedia channels create sensational information by fabricating, exaggerating, distorting, and even spreading rumors to gain the broad attention of the public. Due to a lack of information and judgment, a considerable number of people who do not know the truth can only follow others, and even measure the authenticity of information by the number of times it is transmitted and read. The more times it is transmitted and read, the more inclined people are to believe it. Once people believe the news, they will spread it through their own group networks such as on WeChat or other social media. This makes the news spread to a broader audience.

When this kind of hearsay, especially false information, is spread on a large scale, it can cause social panic. Therefore, relevant departments tend to refute rumors through official media to help people correctly understand the nature of the widely spread but false information and deny such widely dispersed news. In this process, people experience the course of “doubtful” to “convinced” to “do not believe at all,” and their mood undergoes a complete reversal. Meanwhile, official media outlets gain more trust and authority by releasing authoritative information and correcting false information. Official media outlets usually have strict procedures to confirm news sources and information. Refuting rumors and other behaviors further enhances their authority and trustworthiness.

In order to further analyze the different influences that official media and wemedia have on people’s emotions in the process of acquiring information, we further established several models (models 5–8) for the investigation. According to the model results (Table 4), the more people feel that the epidemic situation is worse, the more this stimulates their emotions of panic, and more they feel that the situation is not worse, the less they feel panic. Furthermore, the higher the frequency of their attention, the stronger their emotions of panic, and the lower the frequency, the weaker the panic. Hence, in terms of people who use official media to obtain information, for every unit of increase in their judgment of the prevention and control of the epidemic, their emotions of panic decrease by 0.0186 (−0.0326, −0.149) units, and for every unit of decline in their frequency of attention, their emotions of panic decrease by 0.2012 (0.0362 + 0.165) units. Those who use unofficial media, no matter what their judgments of the COVID-19 situation are, whether severe or not, all feel panic. No matter how often they get information through unofficial media, they all are in a state of panic. This is an important difference between the impact on the emotions of panic caused by the use of different media for information. Combined with the social situation at that time, the outbreak increased rapidly and showed no signs of being contained soon. Under such circumstances, it is quite normal for people to be anxious and even panicky. Even in such a case, people’s approach to and frequency of obtaining information on COVID-19 and their judgment about the developing situation of the outbreak still significantly affect their emotions.

Information obtained from official media and judgments made on it directly influence people’s level of anxiety and public panic. However, unofficial media itself is not a direct influencing factor of people’s emotions. Theoretically, media is only the carrier, and its influence on public emotions is both positive and negative. The decisive factor lies in the nature of the information being transmitted. However, information spread by wemedia is not like that from official media. Information from the official press has both positive and negative functions. However, information obtained from unofficial media only increases people’s anxiety. This implies that during the outbreak period, supervision and control over unofficial media are crucial to curb public panic. Therefore, the use of media has an entirely different effect on people’s emotions by moderating the influence of uncertainty on the feeling of panic. These results confirm our second hypothesis.

Considering the consequences of negative emotions for individuals, the results of model 9 demonstrate that panic has a significant impact on people’s lives (Table 5). For example, the more negative emotions people have, the worse their sleep quality will be. The more anxious they are, the more likely they will stay in a fearful state of mind.

#### 3.2.3. Consequences of Panic

The results of accelerated heartbeat and tension show that although people have a considerable degree of panic due to the outbreak, the panic level can be largely decreased if the government were successful in curbing the outbreak.

The results of model 10 show that if people believe that the government is able to cope with the outbreak effectively and they have full confidence in the government to overcome the outbreak, then even if the outbreak is worsening, this can also relieve their panic to a large extent. This is also consistent with the psychological principle that the more confident people are about something, the more they can remove the uncertainty about their future expectations. This means the governments of different countries should remain fully equipped and confident to combat the new coronavirus.

## 4. Discussion

People’s cognition of the outbreak can be divided into several levels. First is cognition of COVID-19 itself, second is cognition of the consequences caused by COVID-19 (including the spread range), and third is cognition of the government’s and society’s response to the outbreak. Most of the general public’s cognition of COVID-19 comes from their own direct or indirect perception. They strongly believe that it is highly contagious. They do not have any confidence that it can be cured, and thus they are in a state of great uncertainty. With the transmission of the outbreak and the expansion of the spread range, the government has taken decisive measures such as treating patients at designated places, isolating them, closing down cities, etc. The virus not only threatens individuals’ health, but also disrupts the country’s overall development [46,47]. Despite the government’s decisive medical and social countermeasures, how long it will take to conquer the virus is still not clear. Therefore, people are still feeling great uncertainty, and this uncertainty brings anxiety and panic. This is the process by which external stimuli cause people’s initial cognition, the development of the outbreak, and re-cognition, public panic. The panic is mainly due to people’s cognition of great uncertainty developed by the use of media.

Therefore, after the outbreak, the process of acquiring and disseminating information should be carefully monitored by the government. We found that both official and unofficial media play vital roles in public perception of the virus. This finding is partly consistent with a previous study showing that social media plays a vital role during times of disasters [48]. The official media spreads considerable objective information about the characteristics, trends, and resistance of the outbreak, so if the public is more focused on the official media, they can better understand the trends of the outbreak in the right manner. At the height of the outbreak, more people pay attention to it, and they are more likely to panic. Once their attention is reduced, their panic will go down. Therefore, the key variables that affect people’s emotions are not only the channel itself, but also the attention to and cognition of information related to the outbreak.

However, unofficial media play a different role in the process of disseminating information. They also transmit plenty of information about the outbreak, which has varied over time, and it might be because of their loose control over the dissemination. These findings are consistent with recent study findings [49]. Such exaggerated, untrue, and distorted information leads to increased anxiety among people. Therefore, according to the data, when people get information through unofficial media, it will lead to panic whether their attention rises or falls. This is totally different from the role of the official media in the process of disseminating information. Apart from the close relationship between information transmitted by wemedia and people’s panic, wemedia itself may also be a significant cause of the increased panic level. As a result, panic can be generated through the pattern of information construction in addition to the path of external stimulus and cognition.

## 5. Conclusions

This article, through an online questionnaire survey, analyzes the public panic in China after the outbreak of covid-19, as well as the causes and mechanisms of panic and its social consequences.

Our results show that, first, The greater the uncertainty among people, the more panic they feel. From the given cognitive model analysis, we found that the way people have understood the consequences of the prevailing virus has significantly affected their emotions. The more infectious the virus is, the more panicked people will be, and the more uncontrollable people think the virus is, the more they will feel panic.

Second, the influence of uncertainly on the extent of people’s panic is moderated by channels of information. Apart from the close relationship between information transmitted by wemedia and people’s panic, wemedia itself may also be a significant cause of the increased panic level.

### Policy Implications

In order to limit the level of uncertainty among people, the use of unofficial media and sources of incorrect information should be strictly handled during the outbreak time period. Since there are two entirely different paths generating panic, we need to adopt different strategies to deal with panic. For the path of external stimulus and cognition, anxiety and panic are mainly caused by individuals’ uncertainty about the development of the outbreak, its impact, and the response. Therefore, the most important strategy to reduce and alleviate panic is to stabilize people’s expectations and translate their uncertainty into stable expectations. Although it will take time for medical technology to develop treatments for emerging infectious diseases, governments need to demonstrate full confidence and ability to respond to the outbreak in all aspects so that people can have increased confidence that the virus can be controlled in the near future. Only in this way will they tend to cooperate with the government to curb the spread of the virus. Once a society gains confidence, its future expectations will become stable, and uncertainty will be reduced or disappear. Then, people’s panic will be reduced or weakened.

Along the path of information dissemination and social construction, in addition to the panic brought by the outbreak, wemedia itself, as a form of media disseminating information, also aggravates the panic. This kind of panic is unnecessary and can be completely reduced or eliminated. Therefore, in terms of information dissemination during the major outbreak period, the state should regulate the information from unofficial media, and strictly control it. Given the fact that the vast majority of informal media outlets just forward the information without validating the news sources, informal channels should be asked to ensure validity by providing the news sources. If the forwarded information is problematic, the supervision department can ask the original channel of information to correct it. In this way, false news, distorted news, and even rumors can be effectively reduced, thus minimizing the uncertainty and social construction of public panic.

## Figures and Tables

**Table 1:** Exploratory factor analysis of panic.

Variable	Factor 1	Factor 2	Factor 3	Uniqueness
Doubt	0.5361	0.18	0.0583	0.6768
Tension	0.7968	0.3068	−0.0672	0.2665
Worry	0.7484	0.3641	0.0134	0.3071
Helplessness	0.8019	−0.167	0.0163	0.3287
Trepidation	0.8625	−0.2397	−0.059	0.1952
Sadness	0.7211	−0.1222	0.1048	0.4541
Fear	0.842	−0.2193	−0.0303	0.242
Eigenvalue	4.09839	0.40747	0.02375	
Cumulative variance	0.9773	0.9844	0.9861	

**Table 2:** Specific descriptions of dimensions of panic.

Variable	Observations	Mean	SD	Min	Max
Doubt	1539	3.411306	0.963793	1	5
Tension	1539	3.520468	0.962261	1	5
Worry	1539	3.758285	0.938306	1	5
Helplessness	1539	2.954516	1.147966	1	5
Trepidation	1539	2.910331	1.131654	1	5
Sadness	1539	3.157245	1.170716	1	5
Fear	1539	2.933723	1.133839	1	5

**Table 3:** Regression models of cognition, attention, and panic.

Variables	Model 1	Model 2	Model 3	Model 4
High infectivity	0.0373	0.0743 **	0.0797 ***	0.0802 ***
	(0.0269)	(0.0249)	(0.0238)	(0.0239)
Rapid fatality	0.0568	0.0537	0.0419	0.0451
	(0.0314)	(0.0288)	(0.0274)	(0.0274)
High mortality	0.0825 *	0.0372	0.0283	0.0226
	(0.0321)	(0.0296)	(0.0282)	(0.0282)
No specific drug	0.0924 ***	0.0447 *	0.0273	0.0337
	(0.0216)	(0.0201)	(0.0191)	(0.0192)
Personal impact		0.292 ***	0.232 ***	0.232 ***
		(0.0253)	(0.0246)	(0.0246)
Social impact		0.204 ***	0.0847 *	0.0910 *
		(0.0389)	(0.0384)	(0.0383)
Risk (natural and social)		0.0768 ***	0.0610 **	0.0583 **
		(0.0229)	(0.0218)	(0.0217)
Controllable degree		−0.146 ***	−0.116 ***	−0.116 ***
		(0.0262)	(0.0253)	(0.0252)
Attention degree			0.0941 *	0.0888 *
			(0.0387)	(0.0388)
Epidemic situation expansion			0.103 ***	0.103 ***
			(0.0283)	(0.0283)
Information transparency concern			0.0872 ***	0.0889 ***
			(0.0223)	(0.0223)
Adequate life materials concern			0.0982 ***	0.0964 ***
			(0.0210)	(0.0210)
Attention frequency			0.142 ***	0.137 ***
			(0.0265)	(0.0265)
Official media				0.209 **
				(0.0787)
Unofficial media				0.119 *
				(0.0546)
Control variables	Yes	Yes	Yes	Yes
Constant	−0.602	−1.408 *	−1.880 **	−1.991 ***
	(0.614)	(0.591)	(0.589)	(0.588)
*N*	1537	1537	1537	1537
Adj-R^2^	0.066	0.221	0.302	0.308

* *p* < 0.05; ** *p* < 0.01; *** *p* < 0.001.

**Table 4:** Regression models of media use and panic.

Variables	Model 5	Model 6	Model 7	Model 8	Model 10
High infectivity	0.0699 **	0.0707 **	0.0744 **	0.0733 **	0.0677 **
	(0.0243)	(0.0241)	(0.0241)	(0.0243)	(0.0233)
Rapid fatality	0.0863 ***	0.0900 ***	0.0930 ***	0.0886 ***	0.0613 ***
	(0.0197)	(0.0195)	(0.0195)	(0.0197)	(0.0185)
Controllable degree	−0.164 ***	−0.164 ***	−0.165 ***	−0.165 ***	−0.0985 ***
	(0.0257)	(0.0254)	(0.0255)	(0.0257)	(0.0279)
Risk (natural and social)	0.0691 **	0.0738 **	0.0753 ***	0.0701 **	0.0993 *
	(0.0227)	(0.0224)	(0.0225)	(0.0228)	(0.0386)
Attention degree	0.172 ***	0.109 **	0.106 **	0.171 ***	0.0747 **
	(0.0383)	(0.0399)	(0.0400)	(0.0383)	(0.0233)
Personal impact	0.265 ***	0.258 ***	0.260 ***	0.267 ***	0.0600 **
	(0.0255)	(0.0253)	(0.0254)	(0.0256)	(0.0217)
Social impact	0.193 ***	0.192 ***	0.189 ***	0.189 ***	0.104 ***
	(0.0386)	(0.0382)	(0.0382)	(0.0386)	(0.0256)
Prevent and control situation	−0.149 **			−0.152 **	
	(0.0462)			(0.0465)	
Attention frequency		0.165 ***	0.173 ***		
		(0.0273)	(0.0279)		
Prevent and control situation × official media	−0.0326 *				
	(0.0142)				
Attention frequency × official media		0.0362 *			
		(0.0150)			
Attention frequency × unofficial media			0.00672		
			(0.00824)		
Prevent and control situation × unofficial media				−0.00346	
				(0.00773)	
Government’s efficient response					−0.0470 *
					(0.0237)
Official media	0.206 **	0.199 **			
	(0.0684)	(0.0632)			
Unofficial media			0.113 *	0.114 *	
			(0.0482)	(0.0496)	
Control variables	Yes	Yes	Yes	Yes	Yes
Constant	−2.494 ***	−2.177 ***	−2.178 ***	−2.471 ***	−2.689 ***
	(0.623)	(0.588)	(0.590)	(0.624)	(0.631)
*N*	1537	1537	1537	1537	1537
Adj-R^2^	0.239	0.252	0.250	0.236	0.239

* *p* < 0.05; ** *p* < 0.01; *** *p* < 0.001.

**Table 5:** Ordinal logistic models of panic consequences.

Variables	Poor Sleep	Recall the Outbreak Repeatedly	Accelerating Heartbeat and Tension	Recurrent Nightmares
	Model 9a	Model 9b	Model 9c	Model 9d
Panic	0.833 ***	1.170 ***	1.187 ***	0.677 ***
	(0.0560)	(0.0590)	(0.0598)	(0.0565)
Age	0.0553	0.0614	0.0623	0.0161
	(0.0370)	(0.0365)	(0.0363)	(0.0385)
Age^2^	−0.000449	−0.000782	−0.000875	−0.000277
	(0.000545)	(0.000539)	(0.000536)	(0.000570)
Male	−0.0476	0.491 ***	0.169	−0.114
	(0.105)	(0.105)	(0.105)	(0.109)
Junior high school	0.526	3.644 *	0.679	2.559 *
	(1.028)	(1.428)	(1.106)	(1.250)
Senior high school	−0.0596	3.843 **	0.453	2.178
	(0.987)	(1.398)	(1.063)	(1.206)
College	−0.466	3.325 *	−0.142	1.544
	(0.972)	(1.384)	(1.047)	(1.189)
Master’s degree or above	−0.454	3.162 *	−0.245	1.640
	(0.975)	(1.384)	(1.048)	(1.189)
Constant cut1	−0.415	2.129	−0.489	1.441
	(1.105)	(1.493)	(1.182)	(1.344)
Constant cut2	−0.115	2.802	0.0582	1.791
	(1.105)	(1.495)	(1.183)	(1.345)
Constant cut3	1.887	3.988 **	1.796	4.177 **
	(1.106)	(1.496)	(1.184)	(1.348)
Constant cut4	4.192 ***	6.336 ***	4.343 ***	6.135 ***
	(1.113)	(1.500)	(1.188)	(1.361)
*N*	1537	1537	1537	1537

* *p* < 0.05, ** *p* < 0.01, *** *p* < 0.001.

## Supplementary Materials

The following are available online at https://www.mdpi.com/2227-9032/8/3/249/s1, Figure S1: title, Table S1: In the face of Covid-19, the degree of emotional experience you feel, Table S2: After outbreak of covid-19,how you feel.

## References

[1] Sohrabi C., Alsafi Z., O’Neill N., Khan M., Kerwan A., Al-Jabir A., Iosifidis C., Agha R. (2020). World Health Organization declares global emergency: A review of the 2019 novel coronavirus (COVID-19). Int. J. Surg..

[2] Livingston E., Bucher K., Rekito A. (2020). Coronavirus disease 2019 and influenza 2019–2020. JAMA.

[3] Wu F., Zhao S., Yu B., Chen Y.M., Wang W., Song Z.G., Hu Y., Tao Z.W., Tian J.H., Pei Y.Y. (2020). A new coronavirus associated with human respiratory disease in China. Nature.

[4] Imran N., Zeshan M., Pervaiz Z. (2020). Mental health considerations for children & adolescents in COVID-19 Pandemic. Pak. J. Med. Sci..

[5] Haider I.I., Tiwana F., Tahir S.M. (2020). Impact of the COVID-19 Pandemic on Adult Mental Health. Pak. J. Med. Sci..

[6] Brennan J., Reilly P., Cuskelly K., Donnelly S. (2020). Social work, mental health, older people and Covid19. Int. Psychogeriatr..

[7] Hevia C., Neumeyer A. (2010). A Conceptual Framework for Analyzing the Economic Impact of COVID-19 and its Policy Implications.

[8] Rose T.C., Mason K., Pennington A., McHale P., Taylor-Robinson D.C., Barr B. (2020). Inequalities in COVID19 mortality related to ethnicity and socioeconomic deprivation. medRxiv.

[9] Xu X., Zhang L., Chen L., Wei F. (2020). Does COVID-2019 have an impact on the purchase intention of commercial long-term care insurance among the elderly in China?. Healthcare.

[10] Venkatesh A., Edirappuli S. (2020). Social distancing in covid-19: What are the mental health implications?. BMJ.

[11] Evans G.W. (2003). The built environment and mental health. J. Urban Health.

[12] Domínguez-Salas S., Gómez-Salgado J., Andrés-Villas M., Díaz-Milanés D., Romero-Martín M., Ruiz-Frutos C. (2020). Psycho-Emotional approach to the psychological distress related to the COVID-19 pandemic in Spain: A Cross-Sectional Observational Study. Healthcare.

[13] Turner-Musa J., Ajayi O., Kemp L. (2020). Examining Social Determinants of Health, Stigma, and COVID-19 Disparities. Healthcare.

[14] Wang C., Pan R., Wan X., Tan Y., Xu L., Ho C.S., Ho R.C. (2020). Immediate psychological responses and associated factors during the initial stage of the 2019 coronavirus disease (COVID-19) epidemic among the general population in China. Int. J. Environ. Res. Public Health.

[15] Zhang Y., Ma Z.F. (2020). Impact of the COVID-19 pandemic on mental health and quality of life among local residents in Liaoning Province, China: A cross-sectional study. Int. J. Environ. Res. Public Health.

[16] Li S., Wang Y., Xue J., Zhao N., Zhu T. (2020). The impact of covid-19 epidemic declaration on psychological consequences: A study on active weibo users. Int. J. Environ. Res. Public Health.

[17] Adolphs R. (2013). The biology of fear. Curr. Biol..

[18] Darwin C. (1873). The expression of the emotions in man and animals. J. Anthropol. Inst. G.B. Irel..

[19] Gross C.T., Canteras N.S. (2012). The many paths to fear. Nat. Rev. Neurosci..

[20] Davis M., Walker D.L., Miles L., Grillon C. (2010). Phasic vs sustained fear in rats and humans: Role of the extended amygdala in fear vs anxiety. Neuropsychopharmacology.

[21] Mobbs D., Yu R., Rowe J.B., Eich H., FeldmanHall O., Dalgleish T. (2010). Neural activity associated with monitoring the oscillating threat value of a tarantula. Proc. Natl. Acad. Sci. USA.

[22] Lindsay S.J.E. (1984). Treating children’s fears and phobias—A behavioural approach. Behav. Res. Ther..

[23] King N.J., Hamilton D.I., Ollendick T.H. (1988). Children’s phobias: A behavioural perspective. Choice Rev. Online.

[24] Başoǧlu M., ŞalcIoǧlu E., Livanou M.J. (2002). Traumatic Stress Responses in Earthquake Survivors in Turkey. Trauma Stress.

[25] Armfield J.M. (2006). Cognitive vulnerability: A model of the etiology of fear. Clin. Psychol. Rev..

[26] Zhang L., Li H., Chen K. (2020). Effective risk communication for public health emergency: Reflection on the COVID-19 (2019-nCoV) outbreak in Wuhan, China. Healthcare.

[27] Graziano A.M., DeGiovanni I.S., Garcia K.A. (1979). Behavioral treatment of children’s fears: A review. Psychol. Bull..

[28] Wei Y., Tang S.Q. (1998). Several major models of stress theory and their evaluations. J. Psychol. Sci..

[29] Langford I.H., Day R.J., Georgiou S., Bateman I.J. (2000). A Cognitive Social Psychological Model for Predicting Individual Risk Perceptions and Preferences.

[30] Slovic P. (1987). Perception of risk. Science.

[31] Fischhoff B. (1995). Risk perception and communication unplugged: Twenty years of process. Risk Anal..

[32] Xie X.F., Zhou Z.J., Wang L. (2004). Risk behavior characteristics of people with different achievement motivation in risk situations. Acta Psychol. Sin..

[33] Shi K., Fan H.X., Jia J.M. (2003). Risk perception and psychological behavior of Chinese people towards information of SARS. Acta Psychol. Sin..

[34] Sun D.Y. Research on individual and group behavior decision-making under sudden social public crisis. National University of Defense Technology, Changsha, Hunan, China. http://10.1.76.11/kcms/detail/detail.aspx?recid=&FileName=2006127553.nh&DbName=CDFD9908&DbCode=CDFD&uid=M0JlbE8rMDZTZjB0bUU1ekY4alFNRUIxY0hhWERnbTg0NmFYc3dIeGliTnBpQkZk.

[35] Li H.Q., Wang S.H. (2011). Research on the relationship between public demand, risk perception and psychological behavior in sudden disasters. Dajia.

[36] Shao P.R. (2007). Terror from the media? An Introduction and enlightenment of media panic. Shanghai J. Rev..

[37] Wang H. (2003). The causes and elimination of group panic psychology: An interpretation of group psychology from the SARS epidemic. J. Beijing Univ. Posts Telecommun..

[38] Zhou X.H. (2003). Propagation distortion: A social psychological analysis of “SARS” rumors. Sociol. Stud..

[39] Browne K. (2005). Snowball sampling: Using social networks to research non-heterosexual women. Int. J. Soc. Res. Methodol. Theory Pract..

[40] Baltar F., Brunet I. (2012). Social research 2.0: Virtual snowball sampling method using Facebook. Internet Res..

[41] Olive D.J. (2017). Linear Regression.

[42] Su X., Yan X., Tsai C.L. (2012). Linear regression. Wiley Interdiscip. Rev. Comput. Stat..

[43] Hayes A.F., Matthes J. (2009). Computational procedures for probing interactions in OLS and logistic regression: SPSS and SAS implementations. Behav. Res. Methods.

[44] Powers D.A., Xie Y. (2000). Models for ordinal dependent variables. Statistical Methods for Categorical Data Analysis.

[45] Liu X. (2009). Statistical software applications & review ordinal regression analysis: Fitting the proportional odds model using Stata, SAS and SPSS. J. Mod. Appl. Stat. Methods.

[46] Farseev A., Chu-Farseeva Y., Qi Y., Loo D.B. (2020). Understanding economic and health factors impacting the spread of COVID-19 disease. medRxiv.

[47] Gomes da Silva J. (2020). Evolution of COVID19 new cases in 16 countries and Scenarios for Brazil using metaphorical analysis of board, inverted pyramid and papyri. Int. J. Innov. Educ. Res..

[48] Finch K.C., Snook K.R., Duke C.H., Fu K.W., Tse Z.T.H., Adhikari A., Fung I.C.H. (2016). Public health implications of social media use during natural disasters, environmental disasters, and other environmental concerns. Nat. Hazards.

[49] Han X., Wang J., Zhang M., Wang X. (2020). Using social media to mine and analyze public opinion related to COVID-19 in China. Int. J. Environ. Res. Pub. Health.

