# Supplementary material for: Conceptualizing COVID-19 and Public Panic with the Moderating Role of Media Use and Uncertainty in China: An Empirical Framework
Source: Healthcare (Basel). 2020 Aug 2;8(3):249. doi: 10.3390/healthcare8030249 (PMC7551926; doi:10.3390/healthcare8030249)
Supplement: Supplementary file 1 [file healthcare-08-00249-s001.pdf]

Supplementary material

Table S1. In the face of Covid-19, the degree of emotional experience you feel.

	Very Inconsitent	Inconsistent	Neither	Consistent	Very Consistent
doubt	1	2	3	4	5
tension	1	2	3	4	5
worry	1	2	3	4	5
helplessness	1	2	3	4	5
trepidation	1	2	3	4	5
sadness	1	2	3	4	5
fear	1	2	3	4	5

Table S2. After outbreak of covid-19,how you feel.

	Very Inconsitent	Inconsistent	Neither	Consistent	Very Consistent
Poor sleep	1	2	3	4	5
Recall the outbreak repeatedly	1	2	3	4	5
Heartbeats accelerating and tension	1	2	3	4	5
Recurrent nightmares	1	2	3	4	5
